# Targeted delivery of nano-PTX to the brain tumor-associated macrophages

**DOI:** 10.18632/oncotarget.14169

**Published:** 2016-12-25

**Authors:** Lei Zou, Youhua Tao, Gregory Payne, Linh Do, Tima Thomas, Juan Rodriguez, Huanyu Dou

**Affiliations:** ^1^ Department of Biomedical Sciences, Paul L. Foster School of Medicine, Texas Tech University Health Sciences Center El Paso, Texas 79905, USA; ^2^ Graduate School of Biomedical Sciences, Texas Tech University Health Sciences Center El Paso, Texas 79905, USA

**Keywords:** nanoparticles, brain targeted delivery, tumor associated macrophages, glioma

## Abstract

Nanoparticles containing mixed lipid monolayer shell, biodegradable polymer core and rabies virus glycoprotein (RVG) peptide as brain targeting ligand, were developed for brain targeted delivery of paclitaxel (PTX) to treat malignant glioma. RVG conjugated PTX loaded NPs (RVG-PTX-NPs) had the desirable size (~140 nm), narrow size distribution and spherical shape. RVG-PTX-NPs showed poor uptake by neurons and selective targeting to the brain tumor associated macrophages (TAMs) with controlled release and tumor specific toxicity. *In vivo* studies revealed that RVG-PTX-NPs were significant to cross the blood-brain barrier (BBB) and had specific targeting to the brain. Most importantly, RVG-PTX-NPs showed effectiveness for anti-glioma therapy on human glioma of mice model. We concluded that RVG-PTX-NPs provided an effective approach for brain-TAMs targeted delivery for the treatment of glioma.

## INTRODUCTION

The current glioma treatment is completely inadequate indicated by mere one-year average life expectancy after diagnosis [1–3]. A major challenge in delivering drugs is crossing the extremely impermeable blood-brain-barrier (BBB) [4]. Nanotechnology and convection-enhanced delivery [5–7] have been developed in recent years for treatment of glioma, each with highly desirable strengths in certain aspects, but limitations in other aspects. Therefore, while very challenging, the development of an effective brain-glioma targeting delivery system for glioma therapy is much needed [8, 9]. It is particularly difficult to deliver drugs into the brain for anticancer therapy due to poor BBB penetration and inefficiency. RVG interacts specifically with the nicotinic acetylcholine receptor (AChR) on the tight junction of the BBB, between endothelial cells to enable transvascular delivery of RVG-conjugated therapeutics across the BBB to target the brain [10–12]. AChR has recently been reported to be highly expressed in macrophages and brain microglia cells [13, 14]. TAMs play a critical role in glioma tumor development, invasion and growth [15–17]. Remarkably, large proportions of TAMs are strongly correlated with the higher grade of malignancies and poor clinical prognosis in patients [18–21], providing a potential novel target for antitumor treatment.

Lipid and polymeric NPs are two typical drug delivery vehicles for anti-cancer therapy [22–25]. There is thus a natural interest in developing a novel drug carrier that can combine the advantages of both liposomes and polymeric NPs [26–28] to target brain-TAMs. In this work, we synthesized NPs with a mixed lipid monolayer shell and biodegradable polymer core for delivery of PTX. RVG peptide was used as targeting ligand to transport drug-containing NPs across the BBB and preferentially internalized by TAMs [29–31]. Slow release of the potent anticancer agents from NPs engulfed in the TAMs converts the TAMs from a tumor-growth support into a tumor-killing agent. To date, no similar RVG-PTX-NPs approach has been explored [32]. Our study showed that the neural cells excluded RVG-PTX-NPs, which were effectively delivered across the BBB and specifically targeted to TAMs. Thus, targeted delivery by RVG-NPs can be widely used for brain targeted delivery for a variety of purposes, including targeted delivery of PTX to treat glioma.

## RESULTS

### Design of RVG-PTX-NPs

**Figure 1:**
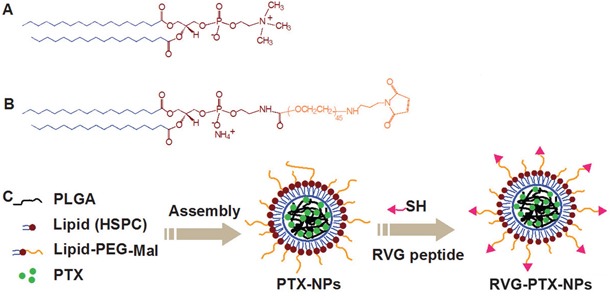
Chemical structure of HSPC **A**. DSPE-PEG-Mal **B**. and the preparation of RVG-PTX-NPs **C**. were schematically illustrated.

The novel PTX-NPs were rationally designed and formed from self-assembly of three biomaterials (modified nanoprecipitation method): (1) poly (lactide-co-glycolide) (PLGA) was selected to form the hydrophobic core due to its ability to encapsulate high amounts of hydrophobic drugs [36–40]. (2) hydrogenated soybean phosphatidylcholine (HSPC), a phospholipid with an appropriate hydrophilic-lipophilic balance (Figure 1A), was chosen to form a monolayer around the hydrophobic core. (3) 1, 2-distearoyl-sn-glycero-3-phosphoethanolamine-N-[Maleimide (polyethylene glycol)-2000] (DSPE-PEG-Mal), a PEGylated DSPE (Figure 1B), was interspersed in the phospholipid monolayer to form a PEG shell. DSPE-PEG-Mal provided electrostatic and steric stabilizations, a longer circulation half-life *in vivo*, as well as functional maleimide (Mal) end groups to specifically react with the thiol groups of HS-RVG (Figure 1C).

### RVG-PTX-NPs characterization

**Figure 2:**
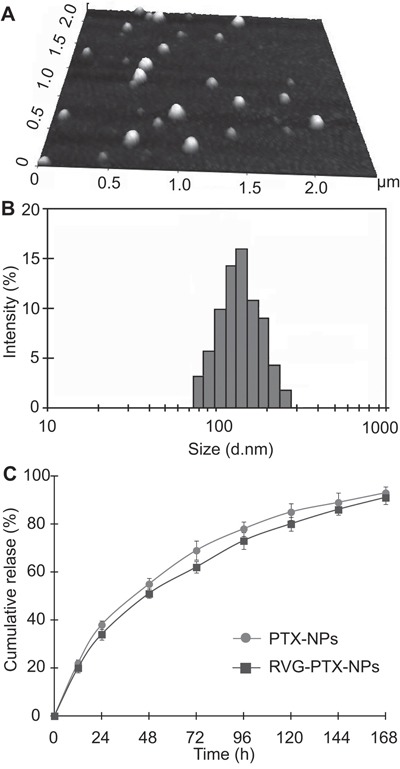
AFM was used to examine the morphology of NPs The images showed that RVG-PTX-NPs had smooth surface and spherical shape. **A**. The size and size distribution of RVG-PTX NPs were analyzed by DLS **B**. The released PTX from PTX-NPs and RVG-PTX-NPs was detected by HPLC **C**. exhibiting slow release profiles.

RVG-PTX-NPs were examined by AFM, DLS, and HPLC [35] to obtain the morphology, size and size distribution, drug loading content, and encapsulation efficiency respectively (Figure 2). AFM and DLS assays demonstrated that the RVG-PTX-NPs were successfully prepared with essentially spherical morphology (Figure 2A) and a mean particle diameter of 139 nm (Figure 2B). PTX-NPs with or without RVG conjugation were further characterized and the particle size, polydispersity index, zeta potential, PTX loading, and encapsulation efficiency were summarized in Table 1. RVG-PTX-NPs were slightly larger than PTX-NPs. The zeta potential of PTX-NPs and RVG-PTX-NPs showed negative charge on the surface.

**Table 1:** Characterization of PTX-NPs and RVG-PTX-NPs

NPs	Particle Size (nm)	Polydispersity index	Zeta Potential (mv)	PTX loading (%)	Encapsulation Efficiency (%)
PTX-NP	137±1.4	0.165±0.012	-50±1.7	1.41±0.15	14.1±1.5
RVG-PTX-NP	139±1.3	0.161±0.017	-47±1.4	1.40±0.13	14.0±1.3

Data represent mean±SE, n=3.

PTX release behavior from NPs was studied *in vitro* in PBS with 0.1% Tween 80 at 37 °C. The cumulative PTX release profiles (Figure 2C) show that there was an initial release of 24.1% in the first 12 h, which could be helpful in suppressing the growth of cancer cells in a short period of time. In the following 72 h, the cumulative release percentage reached 69.8%; and the release proceeded steadily thereafter, which provides the possibility of continuous fight against cancer cells. The cumulative release percentage reached nearly 94% after 7 days, showing the nearly full-release ability of the NP formulation.

### Toxicity of RVG-PTX-NPs

**Figure 3:**
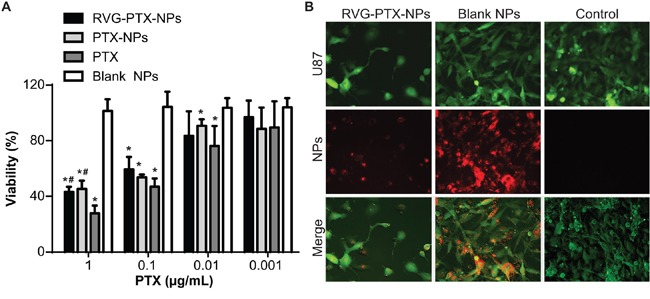
Toxicity of RVG-PTX-NPs RVG-PTX-NPs, PTX-NPs, PTX and blank NPs were treated to U87 at PTX concentration of 0.001 to 1 μg/ml for 72 hours. Dose dependent toxicities of RVG-PTX-NPs, PTX-NPs and PTX were analyzed by MTT assay **A**. Fluorescence microscopy analysis showed the cytopathological changes of U87 cells (**B, green**) after 24 hours exposure to Rhodamine 6G labeled RVG-PTX-NPs and blank NPs (**B, red**). The untreated U87 cells served as control.

In order to determine tumor-specific toxicity, we treated U87 cells with RVG-PTX-NPs, PTX-NPs and PTX at concentrations of 0.001, 0.01, 0.1 and 1 μg/mL for 3 days. The blank NPs served as control (equal PLGA concentration). The dose-dependent anti-glioma activities were detected in RVG-PTX-NPs, PTX-NPs and PTX treated groups, while blank NPs did not exhibit toxicity at each time points (Figure 3A). RVG-PTXNPs and PTX-NPs showed half maximal inhibitory concentration (IC_50_) at concentrations as low as 0.1 μg/mL. RVG-PTX-NPs and PTX-NPs showed significant toxicity (p< 0.05) to U87 cells and no significant differences between the two formulations (Figure 3A). At same concentration, the toxicity of PTX was greater than PTX-NPs and RVG-PTX-NPs due to the slow release process.

GFP transfected U87 cells were treated with red fluorescence labeled RVG-PTX-NPs and blank NPs for 24 hours. The untreated GFP-U87 cells served as control. The anti-cancer activity of RVG-PTX-NPs was studied by cytopathology and morphology analysis (Figure 3B). RVG-PTX-NP significantly decreased the numbers of U87 cells and altered the morphological features. The results showed that RVG-PTX-NPs kill U87 cells and inhibit the proliferation. In contrast to similar uptake of blank NPs, no obvious toxicity was detected in U87 cultures. This study confirmed that the designed RVG-PTX-NPs provide high biocompatibility of core-lipid shell structure.

### RVG-PTX-NPs selective targeted to BMM

**Figure 4:**
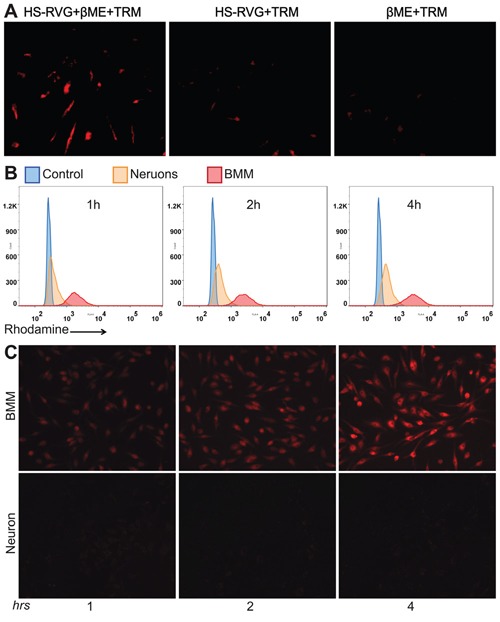
To test RVG specific binding to macrophages, we made red fluorescence labeled HS-RVG through directly reaction with TRM (HS-RVG+TRM), HS-RVG reduced by βME before reaction with TRM (HS-RVG+βME+TRM), and βME reaction with TRM without HS-RVG (βME+TRM) BMM were treated to HS-RVG+TRM, HS-RVG+βME+TRM and βME+TRM for 2 hours. The specific binding showed in BMM treatment with (HS-RVG-βME-TRM (**red**). No specific binding signals seen in cultures treated to HS-RVG-TRM or βME+TRM **A**. RVG-PTX-NPs were incubated with BMM and neurons for 1, 2 and 4 hours. Flow cytometry was used to determine fluorescence signals when RVG-PTX-NPs uptake by BMM and neurons **B**. The fluorescence microscope was used to visualize intracellular distribution of RVG-PTX-NPs in BMM and neurons **C**. The significant increases of red signal were observed in BMM. No red fluorescence was detected in neurons at any time points.

RVG peptide specifically binding to AChR is a key step for rabies virus to cross the BBB and infection of the brain. We first incubated fluorescence labeled RVG peptide with BMM. Fluorescence labeled RVG was obtained through the optimized reaction procedure of HS-RVG with TRM. After 1 hour incubation of fluoresce labeled RVG, BMM exhibited density of red fluorescence (Figure 4A). Only very weak fluorescence signals were observed in the experiment groups due to unspecific binding. No fluorescence was obtained from the control groups. This experiment further verified the specific binding of RVG peptide to AChR. We came to the conclusion that our BMM has the expression of AChR; and it is better to reduce disulfide bond; which occur during the storage of HS-RVG before the additional reaction to maleimide group.

In order to detect the nanoparticle inside the cells, we labeled the nanoparticle with “red fluorescence” Rhodamine 6G by encapsulating the dye inside the nanoparticle. BMM and the hippocampal neurons were incubated with RVG-PTX-NPs for 1, 2 and 4 hours. Flow cytometry assay was used to determine the levels of fluorescence RVG-PTX-NPs uptake by BMM and the hippocampal neurons. In contrast to poor fluorescence signals in the hippocampal neurons, a significant uptake by BMM was seen at all the time points with a very apparent shift to higher intensity (Figure 4B).

To test if RVG conjugated NPs at size of 140 nm facilitated BMM uptake but “stopped” the neuronal uptake, BMM and the hippocampal neurons were treated with RVG-PTX-NPs at same concentration. Microscopic images were taken at 1, 2 and 4 hours post-treatment. RVG-PTX-NPs entered BMM very quickly (Figure 4C, upper panel). At four hours, almost 100% of BMM contained greater levels of “red” RVG-PTX-NPs. In contrast, poor uptake of RVG-PTX-NPs by neurons was obtained at all the time points (Figure 4C, lower panel). The results confirmed that RVG-PTX-NPs at size of 140 nm were selectively taken up by BMM but not by neurons, leading to a specific brain-TAM targeted delivery system. Moreover, RVG-NPs did not bind to the neuronal axons (nerves) and dendrites, avoiding potential peripheral neurotoxicity. No significant increase of fluorescence was observed after 4 h of RVG-PTX-NPs treated to BMM (data not shown). With increased incubation time, the gradually increased fluorescence intensity indicated a time-dependent uptake pattern leading to greater intracellular levels at 4 hours.

In addition, quantitative investigation has been conducted by HPLC to measure the levels of PTX inside the BMM. With the increase of incubated time from 1, 2, and 4 to 8 hours, the intracellular amount of PTX were increased in a time-dependent manner. The results were well in accordance with the fluorescence images. With initial 1.5 μg of PTX treated to BMM, the intracellular PTX reached to a maximum amount of 0.65 μg in PTX-NPs group and 0.75 μg in RVG-PTX-NPs groups, respectively. In contrast to PTX-NPs, the relative higher intracellular levels of PTX presented in RVG-PTX-NPs treated BMM.

### RVG-PTX-NPs transportation by BMM

**Figure 5:**
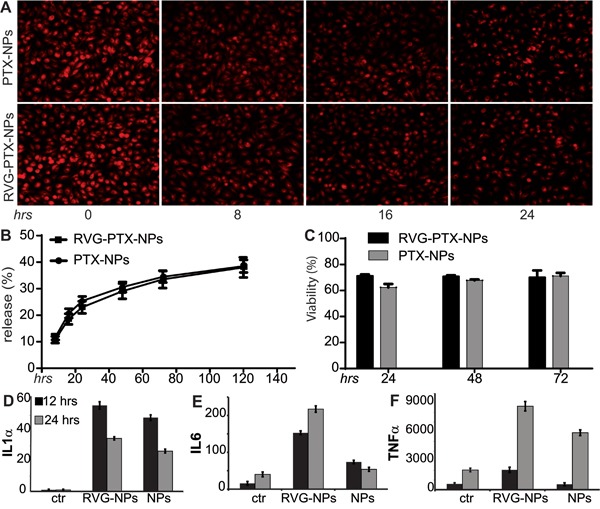
Maximum uptake of RVG-PTX-NPs and PTX-NPs by BMM was counted as experimental time “0” (A, hrs 0) for the release study under fluorescence microscope Images show that BMM release PTX/PTX-NPs led to loss of red signals following each medium change every 8 hours. HPLC assay was used to detect extracellular PTX in medium (released PTX) and intracellular PTX in cell lysates. The percentage of PTX content released from BMM to the medium showed in **B**. MTT assay was used to detect that PTX loaded NPs did not cause BMM death **C**. TAMs treated with RVG-PTX-NPs and PTX-NPs caused alterations in cytokine profiles of IL1α **D**., IL6 **E**. and TNFα **F**. levels (*p*<0.05). All data were acquired from triplicate experiments.

The critical step after BMM has taken up RVG-PTX-NPs is to transport these intracellular particles to neighboring glioma cells. First, we examined the ability of BMM to release PTX-NPs or PTX. The release profile was depicted by means of both the intensity of fluorescence remaining in the BMMs, and the amount of PTX traveled into the medium. After maximum uptake of RVG-PTX-NP by BMM for 4 hours, fresh medium was changed every 8 hours with immediate fluorescence images captured. BMMs released fluorescence labeled RVG-PTX-NPs and PTX-NPs in a sustainable manner (Figure 5A), and greater “red signals” were still remaining inside the BMM even after 24 hours.

To identify the release profile, BMMs were treated with RVG-PTX-NPs at the initial concentration of 0.01 mg/mL PTX for 4 hours. Then, the supernatant was collected and the fresh medium was added at each time point. PTX was consistently released up to 5 days (Figure 5B). Importantly, the total amount of 40% PTX was released from BMM. The release profiles depicted by fluorescent and PTX levels had a slight difference due to the “red signal” attached to NPs but not PTX.

Next, we studied whether RVG-PTX-NPs toxic to BMM. RVG-PTX-NPs and PTX-NPs were treated to BMM at the same concentration. The MTT assay showed a reduction of 25~36% in BMM viability (Figure 5C).

### RVG-PTX-NPs induced TAMs polarization

RVG-PTX-NPs and PTX-NPs induced TAMs polarization were evaluated by qRT-PCR. As expected, the expression of IL1α (Figure 5D), IL6 (Figure 5E), and TNFα (Figure 5F) were significantly increase in RVG-PTX-NPs treated TAMs at 12 and 24 hrs as compared to control. In contrast, PTX-NPs induced greater levels of IL1α, and IL6 at 12 hrs and TNFα at 24 hrs. Notice, gene expression of IL1α, IL6 and TNFα were increased to 25, 10 and 3 folds at 12 and 17, 5 and 4 folds at 24 hrs in RVG-PTX-NPs compared to 19, 5 and 1 fold at 12 hrs and 13, 1 and 3 folds at 24 hrs in PTX-NPs.

### Anti-glioma activities of RVG-PTX-NPs packaged BMM

**Figure 6:**
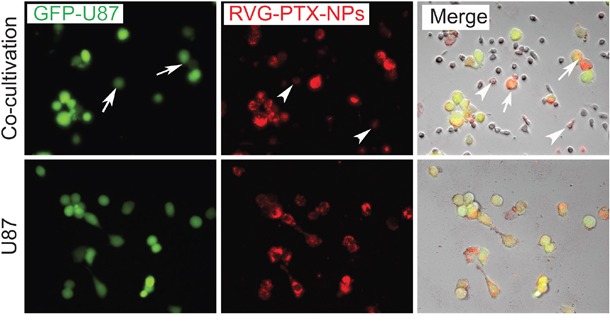
Cytotoxicity was analyzed in RVG-PTX-NPs pre-loaded TAMs (RVG-PTX-NPs-TAMs) co-cultivation with U87 In the co-cultivation system, RVG-PTX-NPs-TAMs (**red**, arrow head) were co-cultured with GFP-U87 (**green**,) for 24 hours (**upper panel**). Imaging showed RVG-PTX-NPs (red) within TAMs were transported into U87 cells (**green**, arrow), identifying as yellow-red (**merge**, arrow). U87 directly treated with RVG-PTX-NPs served as control (**lower panel**). In contrast, RVG-PTX-NPs-TAMs provided a significant transportation and greater toxicity to U87.

In order to verify the effectiveness of the BMM based delivery system, green fluorescence labeled GFP-U87 cells (Figure 6, green) were co-cultured with RVG-PTX-NPs pre-loaded BMM (RVG-PTX-NPs-BMM, Figure 6, red). An equal amount of RVG-PTX-NPs were directly treated to GFP-U87 cultures to serve as the control. Microscopy images showed that RVG-PTX-NPs-BMM transporting “red particles” into GFP-U87 cells showed as yellow (Figure 6). A greater level of “red” RVG-PTX-NPs was determined in GFP-U87 cells after 24 hours of co-cultivation. As a result, the morphology of the U87 cell became irregular with significant loss of the cell numbers. This phenomenon obviously indicated the high efficiency of RVG-PTX-NPs-BMM delivery system via cell-to-cells transportation.

### RVG-PTX-NPs mediated trans-BBB delivery

**Figure 7:**
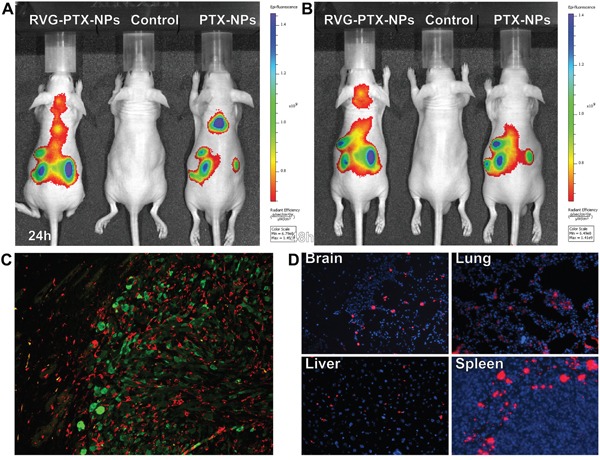
*In vivo* images were taken at 24 **A**. and 48 **B**. hours in live animals after administration of DiSC_3_(5) labelled RVG-PTX-NPs to mice via tail vein. Significant amount of fluorescence signals appeared in the head after 24 and 48 hours. The brain sections from human glioma of SCID mouse model were labeled with IBa-1 and GFP to identify TAMs/microglia (**C**, red) and human glioma U87 cells (**C**, green). The frozen sections was mounted with DAPI (blue) to examine the tissue distribution of DiSC_3_(5) labelled RVG-PTX-NPs (**D, red**).

*In vivo* real-time imaging analysis was used to investigate whether RVG provided trans-BBB delivery for brain TAMs targeting in an intact BBB [10, 41, 42]. DiSC_3_(5) labeled RVG-PTX-NPs and DiSC_3_(5) labeled PTX-NPs were administered via a tail vein. The untreated mice served as the control. *In vivo* fluorescent images showed that fluorescence signals significantly increased in the brain of RVG-PTX-NPs treated mouse at 24 hours (Figure 7A) and 48 hours (Figure 7B). In contrast to poor brain distribution of PTX-NPs, RVG-PTX-NPs facilitated across the BBB and accumulated into the brain area.

Histology assay was used to study glioma growth inducing tumor-associated macrophages/microglia (TAMs) activation. A human glioma of mouse model was developed by inoculation of GFP-U87 cells into the SCID mouse. Double immunostaining of the brain sections illustrated Iba-1+ TAMs (red, Figure 7C) were observed accumulation in GFP+ U87 tumor mass (green, Figure 7C). Morphological differences of Iba-1+ TAM and microglia were observed between inside of the tumor and outside area. To best visualize red fluorescence labeled RVG-PTX-NPs targeted delivery to macrophages, fresh-frozen tissue sections were mounted with DAPI mounting medium. The red fluorescence labeled RVG-PTX-NPs were detected from the brain, lung, liver and spleen sections (Figure 7D). The red fluorescence RVG-PTX-NPs were firmly targeting to the macrophages located in red pulp of the spleen, and epithelium surface of the liver and the lung. Importantly, the red fluorescence spots in the brain section confirmed the trans-BBB capability and macrophages-targeted delivery of RVG-PTX-NPs.

### RVG-PTX-NPs preventing tumor progression

**Figure 8:**
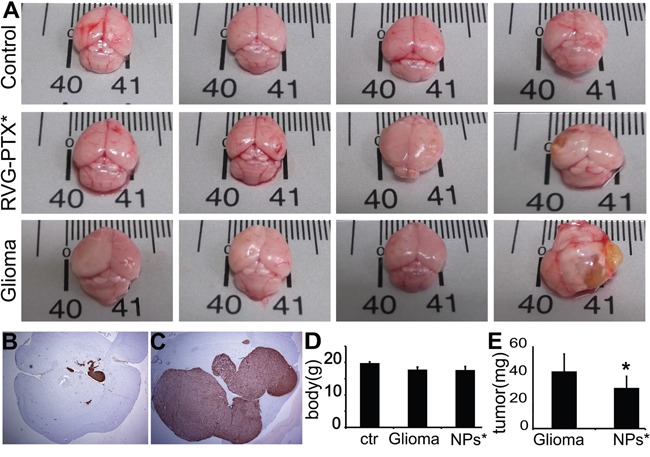
Mice were sacrificed at 4 weeks to evaluate the brain size and weight, and histopathological changes In contrast to normal mouse (**A, upper panel**), untreated glioma mice (**A, lower panel)** exhibited tumor growth, largely increasing the brain size. Tumor growth was prevented by RVG-PTX-NPs (**A**, **middle panel**), leading to comparable brain size to the normal control. The brain sections were histopathologically evaluated by antibody to GFP to identify the GFP-U87 cells (**B and C, brown**). Representative images revealed significant prevention of U87 growth in RVG-PTX-NPs treated glioma mice (B) compared to untreated group (C). The body **D**. and tumor **E**. weight were measured to determine the body loss and tumor growth for evaluation of the anti-glioma efficacy.

The anti-glioma efficacy of RVG-PTX-NPs was determined on human glioma of mice model by inoculation of GFP-U87 cells into the SCID mouse. The brains from normal animals, untreated glioma mice and RVG-PTX-NPs treated glioma mice were compared to assess the size of brain with tumor growth. In contrast to normal control (Figure 8A, upper panel), the glioma mice showed increased brain size with tumor growth (Figure 8A, low panel). Administration of RVG-PTX-NPs caused significant prevention of tumor progress, leading to comparable brain sizes (Figure 8A, middle panel). Next, anti-glioma efficacy was histopathologically evaluated using antibody to GFP to label GFP-U87 in the mouse brains. Low magnification (2X) images of RVG-PTX-NPs treated glioma mice revealed that GFP-U87 cells formed small clusters in the brain (Figure 8B). The tumors grew to a large mass (Figure 8C) in glioma mice without treatment. To better evaluate the anti-glioma efficacy of RVG-PTX-NPs, the body and tumor weight were assessed. The body weight was significantly lost in glioma mouse compared to normal control. The body weight of glioma mice with or without RVG-PTX-NPs treatment did not exhibit significant differences (Figure 8D). Importantly, tumor growth was significantly reduced in RVG-PTX-NPs treated mice (Figure 8E) as compared to untreated group. The results provided strong evidence that RVG-PTX-NPs are capable of cross the BBB and effective for anti-glioma therapy.

## DISCUSSION

The close correlation of TAMs and poor clinical outcome in patients [18–21] offers a novel targeting strategy for treatment of glioma [17, 43–46]. PTX has demonstrated effective anti-cancer activities in the treatment of a number of cancers, including glioma [47, 48]. The difficulty in clinical application is due to poor solubility and an inability to cross the BBB [49, 50]. Peptide conjugates for targeting delivery has the highly desirable advantages of receptor-mediated specificity. A short RVG peptide was found to have the ability to cross the BBB [51, 52]. Very recently, RVG has been shown to bind specifically to macrophages via highly expressed AChR on cells surface [11, 14, 53]. However, cell selective targeting has substantial challenges. We overcome these challenges in three steps to 1) cross the BBB to target the brain 2) stop RVG-AChR binding in neurons to prevent neurotoxicity and 3) selectively target TAMs for transportation of drugs to glioma.

The first challenge is to cross the BBB to target the brain. RVG interacts specifically with the AChR on the tight junction of the BBB between endothelial cells to deliver RVG-PTX-NPs across the BBB to target brain-TAMs. Through the conjugation of RVG on the surface of PTX-NPs, we demonstrated a greater accumulation of RVG-PTX-NPs in the brain than PTX-NPs. For brain-targeted delivery, the size distribution is generally limited to ~200 nm in diameter [54, 55]. A parallel study of physiological properties of RVG-PTX-NPs alter ligand-receptor mediated TAMs targeting delivery further addressed the mechanism of single-cell specificity [56]. We developed a favorable size of RVG-PTX-NPs to improve poor solubility and BBB penetration and to target TAMs. In this formulation, polymeric core was used to encapsulate the hydrophobic PTX. During the assembly process, lipid formed as the shell of the nanoparticle. The application of DSPE-PEG-Mal PEGylated the shell, and thus, elongated the circulation time of the nanoparticle by preventing their rapid uptake and removal form body circulation by the reticuloendothelial system. More importantly, the DSPE-PEG-Mal readily provided the mounting site for RVG. Through the self-assembly process and highly efficient surface modification reaction, PTX was encapsulated inside and RVG was successfully conjugated on the surface. The well formulated RVG-PTX-NPs led to higher PTX encapsulation, consistent release, negative charge, biomodified surface, and controlled size. The surface properties of RVG-PTX-NPs significantly affected nanobiological behaviors *in vitro* and *in vivo*.

*The second challenge* for brain targeted delivery by RVG-conjugates is to interrupt RVG-AChR binding in the neuron. Targeting to non-phagocytic cells, such as neurons, must be small enough to “hold”” the particles on the cell's surface for further uptake process. We prepared RVG-PTX-NPs with relevant “bigger” size at ~140nm. Our results demonstrated that of the size controlled RVG-PTX-NPs interrupted the physical balance of RVG-AChR binding, and led to the elimination of uptake by neurons. The neurotoxicity of PTX can be reduced, as we demonstrated that the uptake of NPs by neurons is negligible.

The third challenge was to target TAMs for subsequently transporting PTX-PTX-NPs to neighbor glioma cells. In pathological condition, the macrophages are under very active processes of phagocytosis and exocytosis. Macrophages offered the dual benefactions of RVG-AChR binding and larger NPs favorable uptake, leading to selective and specific targeting to the brain-TAMs. Importantly, co-cultivation of RVG-PTX-NPs-TAMs and U87 cells exhibited a greater intracellular levels of “red” RVG-PTX-NPs in U87, confirming the effective cell-to-cell transportation. TAMs targeted delivery of RVG-PTX-NPs led to the control release of PTX and a significant cell-to-cell direct transportation nanoparticle from TAMs to U87 cells. The results from *in vitro* and *in vivo* studies indicated that the biological functions of macrophages facilitated uptake of size-controlled RVG-PTX-NPs by receptor-mediated phagocytosis, and transported PTX to kill U87 cells via exocytosis.

Glioma TAMs are playing a major role in the creation of a local tumor microenvironment that is immunosuppressive and promotes glioma growth [43, 57–59]. Polarization of immunosuppressive M2 TAMs toward activated inflammatory M1 macrophages is critical for TAMs targeting delivery to treat glioma. The pro-inflammatory cytokines IL-6 and TNF-α have been known for their ability to drive TAMs toward the M1 phenotype [60, 61]. As our data suggest, TAMs treated with RVG-PTX-NPs and PTX-NPs show the increase of IL-6 and TNF-α as compared to untreated TAMs, indicating that both NPs can effectively polarize TAMs to M1 phenotype. The possible explanation for this may be due to the fact that RVG-PTX-NPs and PTX-NPs are able to induce TAMs activation, increased pro-inflammatory cytokines and predominant M1 polarization. In addition, the internalized RVG-PTX-NPs and PTX-NPs and the release of PTX can activate pro-inflammatory cytokine signaling pathways. The increase in IL-6 and TNF-α, leads to a re-education of TAMs from M2 to an M1 like phenotype, suggesting a radical shift of the TAMs from pro-tumorigenic to an anti-tumorigenic nature when they are treated with RVG-PTX-NPs and PTX-NPs.

In addition, RVG-PTX-NPs provide an effective vehicle to transport anti-glioma drug across the BBB to target TAMs. The brain-TAMs targeted delivery of RVG-PTX-NPs offered a significant anti-glioma efficacy *in vivo*. The functional alteration of TAMs will attenuate tumor-promoting secretions and inhibit the invasion and growth of glioma. The RVG-NPs TAMs targeting delivery system offers a potential immunotherapy strategy for anti-glioma therapy. The mechanism of NPs uptake, release and transfer to tumor cells by macrophages remains unclear. Compared to PTX-NPs, the accumulation of RVG-PTX-NPs in the brain leads to the fundamental questions of improving pharmacokinetics and achieving single-cell targeting. Taking advantage of highly developing nanotechnology, we are now able to alter the composition, size, shape, and surface property of a rationally designed nanoformation, allowing the incorporation of different drugs or molecular probes with a broad range of physical and biochemical properties.

## MATERIALS AND METHODS

### MATERIALS

Poly(D,L-lactide-co-glycolide) (50/50) with terminal carboxylate groups (PLGA; inherent viscosity, 0.55-0.75dl/g in hexafluoroisopropanol; MW:~ 44 kDa) was obtained from Absorbable Polymers International (Pelham, AL, USA). Hydrogenated L-α-phosphatidylcholine (Soy) (HSPC, MW: 762) and DSPE-PEG-Mal (1,2-distearoyl-sn-glycero-3-phosphoethanolamine-N-[maleimide(polyethylene glycol) -2000]) were obtained from Avanti (Alabaster, AL). The peptide RVG with cysteine at the C-terminus (YTIWMPENPRPGTPCDIFTNSRGKRASNGGGGC, HS-RVG) was synthesized by RS Synthesis (USA) Ltd. Paclitaxel (PTX), Rhodamine 6G (Sigma-Aldrich), β-Mercaptoethanol (βME) and tetramethylrhodamine-5-maleimide (TRM) was obtained from Sigma-Aldrich and 3,3'-Dipropylthiadicarbocyanine Iodide (DiSC_3_(5) was from Molecular Probes.

Bone marrow cells were isolated from the femur of Balb/C mouse and differentiated to macrophages with 500-1000 U/mL macrophage colony stimulating factor (MCSF). Human glioma U87 cells and embryonic mouse hippocampal neurons were purchased from ATCC. The U87 cells were transfected with green fluorescence (GFP-U87) to appear green fluorescence signals. For fluorescence microscopy, flow cytometry, and uptake and release experiments cells were collected from six-well plate with around 80% confluency. For MTT assay, cells were collected from ninety-six-well plate with around 80% confluency.

### Preparation of RVG-PTX-NPs

PTX-NPs were synthesized using a modified nanoprecipitation technique combined with self-assembly, as described previously [26, 33]. PLGA, PTX (10% of the weight of PLGA) and Rhodamine 6G (2% of the weight of PLGA) was dissolved in the organic solvent DMF at a PLGA concentration of 8 mg/mL. HSPC and DSPE-PEG-Mal (8:1, molar ratio) were dissolved in a 4% ethanol aqueous solution at 60% PLGA polymer weight and heated to 65°C. The PLGA/DMF solution was then added drop-wise into the preheated lipid aqueous solution (1 mL/min) under gentle stirring followed by vortex for 3 minutes. The NPs were allowed to self-assemble for 2 h with continuous stirring at room temperature. NPs stock solution was obtained at the concentration 15 mg/mL. From *in vivo* imaging, RVG-PTX-NPs were labeled with DiSC_3_(5) instead of Rhodamine 6G during the preparation.

HS-RVG (1 mg/mL) was reduced by βME (5 equal molar amounts) in aqueous solution for 4 hours and PTX-NPs (15 mg/mL) was then added for Mal groups of the PTX-NPs reaction with thiol groups of HS-RVG to 24 hours at room temperature in dark [34]. In this reaction, the feed ratio between overall thios (HS-RVG plus βME) and maleimide was fixed as 1:1.

### Characterization

The particle size, size distribution, charge and morphology were measured by Malvern Zetasizer Nano ZS dynamic light scattering (DLS) and atomic force microscopy (AFM). The drug encapsulation efficiency (EE, %) was calculated as: (actual amount of drug encapsulated)/(initial amount of drug used in the fabrication) × 100. The drug loading fraction (DL, %) was calculated as: (weight of PTX in NPs/weight of the NPs) × 100.

### Reversed phase high performance liquid chromatography (RP-HPLC)

For HPLC analysis, PTX extracted from the culture medium containing nano-PTX, the cell lysate and nanosuspension were added to autosampler vials with glass inserts. The levels of PTX were determined by RP-HPLC using a Phenomenex Luna C18 column isocratically eluted with a 60:40 v/v acetonitrile/water. The system was run at a flow rate of 0.8 mL/min, and PTX was detected at 227 nm. The concentration of PTX in the solution was determined by the peak area using a standard curve obtained with fixed concentrations of PTX in acetonitrile (correlation coefficient of R2 =0.9965). The limit of detection was 0.06 μg/ml. The relative standard deviation (RSD) of the content of paclitaxel in the intra-day and inter-day (five days) was less than 2%. [35]

### RVG-PTX-NPs stability

PTX release from the cargo was used to analyze the stability of RVG-PTX-NP. The suspension of RVG-PTX-NPs and PTX-NPs were placed into a 1 mL dialysis membrane tubing (MWCO = 3500, Spectrum Laboratories, Inc.) and dialyzed against 100 mL PBS with 0.1% Tween 80. At experimental time points, 2 mL of the supernatant containing released PTX was collected and determined by RP-HPLC.

### Bone marrow derived macrophages (BMM), TAMs and U87 cells cultures

Using a protocol approved by the Institutional Animal Care and Use Committee (IACUC), mouse (Balb/C, male 4-6 weeks) femur bone marrow was dissociated into single-cell suspensions. Bone marrow cells were cultured in DMEM with fetal bovine serum (10%), penicillin, streptomycin, and 500 U/ml. Macrophage-colony stimulating factor (MCSF) is not only an important regulator of macrophage production, but also stimulates macrophages/monocyte polarization into TAMs under pathological condition. TAMs are believed to represent proliferation of macrophage progenitor cells within the tumor microenvironment. Bone marrow cells were supplied with MCSF or plus co-stimulation by glioma U87 or cultured medium to differentiate into BMM or TAM. Human glioma U87 cells labeled with green fluorescence (GFP-U87) and embryonic mouse hippocampal cell lines (neuron cells, mHippoE-14) were cultured in DMEM supplemented with 10% fetal bovine serum.

### Fluorescence microscopy

Fluorescence microscopy (Nikon Instruments Inc., Melville, NY) was used to examine intracellular distribution of Rhodamine 6G labeled RVG-PTX-NPs and PTX-NPs in BMM, neurons and U87 cells.

### Flow cytometry

BMM and neurons were treated to fluorescence labeled RVG-PTX-NPs for 1, 2 and 4 hours. The cells were fixed with 2% paraformaldehyde and analyzed by Flow cytometry (Gllios 10-color flow cytometer, Beckman Coulter, Inc., Brea CA).

### MTT assay

MTT reduction was used to measure RVG-PTX-NPs and PTX-NPs induced cytotoxicity. Briefly, each well (96-well plate) was replaced by 100 μL of MTT solution and incubated for 45 minutes at 37 °C. At the end of the incubation period, 100 μL of dimethyl sulfoxide (DMSO) was replaced to react for 15 minutes at room temperature. The plates were assessed by microreader with maximum absorbance at 490 nm.

### Uptake and release

For uptake experiments, BMM and neurons were incubated with medium containing RVG-PTX-NPs or PTX-NPs at a concentration of 0.5 mg/mL (calculated as PLGA) for 1, 2 and 4 hours. The cells were washed three times with PBS. The fluorescence signals were analyzed by fluorescence microscopy and flow cytometry. Quantitation of the intracellular PTX by RP-HPLC was performed at 1, 2, 4 and 8 hours after BMM incubation with RVG-PTX-NPs and PTX-NPs at a concentration of 0.01 mg/mL (calculated as PTX).

For BMM release experiments, at the end of 4 hour's uptake, the cultures were washed and fresh medium was replaced every 8 hours to 24 hours, following total medium changes daily. Fluorescence images were taken during each medium change by fluorescence microscopy. The medium containing released PTX from BMM were analyzed by HPLC, and the released PTX was calculated by percentage of extracellular (medium) and intracellular (BMM) PTX levels.

### Human glioma of mouse model and RVG-PTX-NPs treatment

Four-to-six-month old, male SCID mice were used to develop a mouse model of human glioma under strict observance of the Texas Tech University Health Sciences Center, Institutional Animal Care and Use Committee (IACUC). Briefly, the animal was secured with ear-bars and mouth-piece on a stereotactic apparatus. Intracerebral injection of GFP-U87 cells (5 × 10^5^) was performed by 10 μl microvolume syringe with coordination at: 0.3-0.6 mm posterior to bregma, 3.5 mm lateral from the sagittal midline, and at a depth and angle of 3.6 mm and 35^0^ from the vertical line.

Anti-glioma treatment was performed at 3 days post intracerebral injection, allowing recovery from the surgery. The glioma mice were grouped to untreated and RVG-PTX-NPs treated groups (7 mice/group). The mice without U87 inoculation served as normal control. RVG-PTX-NPs treated mice (0.5 mg per injection) were weekly administered via tail vein. Mice were sacrificed at 4 weeks for anti-glioma evaluation.

### Living animal imaging

DiSC_3_(5) labeled RVG-PTX-NPs and PTX-NPs were administered to mice via tail vein. Live animal imaging was performed at 24 and 48 hours by IVIS Lumina System (Xenogen Corp., Alameda, CA). Images were taken and coupled to Living Image software for data acquisition.

### Histology

The brain, liver, lung, and spleen were collected and cut to 5 um sections to stain with DAPI (4',6-diamidino-2-phenylindole, blue). Double-labeling fluorescence images were used to identify the tissue distribution of red RVG-PTX-NPs. Immunohistochemistry was used to identify U87 cells (brown) in the brain section by using antibody to GFP.

### Quantitative real time PCR (qRT-PCR)

The cytokine RNA levels of IL6 and TNF-α were analyzed by qRT-PCR to detect the polarization of RVG-PTX-NPs treated TAMs. Total RNA from BMM was isolated and measured using a Nanodrop. The primers and probe are: (1) GAPDH as forward primer: CGGCCGCATCTTCTTGTG, reverse primer: ACACCGACCTTCACCATTTTG, and probe: AGTGCCAGCCTCGTCCCGTAG; (2) IL6 as forward primer: CTGCAAGAGACTTCCATCCAGTT, reverse primer: AGGGAAGGCCGTGGTTGT, and probe: CCTTCTTGGGACTGATGCTGGT; and (3) TNF-α as forward primer: GGCTGCCCCGACTACGT, reverse primer: ACTTTCTCCTGGTATGAGATAGCAAAT, and probe: TCCTCACCCACACCGTCAGCC. The reverse primer was used to make cDNA from RNA that was further amplified using primers and probe at 50°C for 2 min, 95°C for 10 min, and 40 cycles at 95°C for 15 sec and 60°C for 1 min. DNA from 8E5 cells, which contain one integrated copy of proviral DNA per cell, was used to prepare the standard curve. Separate GAPDH amplifications were used as an endogenous control to ensure that equal amounts of RNA were used. Results are expressed as normalized mean copy number ± SEM.

### Statistical analyses

One-way analysis of variance was used to compare the treatment groups for each of the outcomes. Residual plots were examined to check whether the model assumptions were met. If the model assumptions were not met, then the log_10_ or square root transformation was applied to help with model fit. If the overall F-test was statistically significant, then pairwise comparisons were conducted, with adjustments made for multiple comparisons, using Tukey's method. P values less than 0.05 were considered to be statistically significant.
